# Normalized skin conductance level could differentiate physical pain stimuli from other sympathetic stimuli

**DOI:** 10.1038/s41598-020-67936-0

**Published:** 2020-07-02

**Authors:** Satomi Sugimine, Shigeru Saito, Tomonori Takazawa

**Affiliations:** 1 Intensive Care Unit, Gunma University Hospital, 3-39-15 Showa-machi, Maebashi, Gunma 371-8511 Japan; 2 Department of Anesthesiology, Gunma University Graduate School of Medicine, 3-39-22 Showa-machi, Maebashi, Gunma 371-8511 Japan

**Keywords:** Neurophysiology, Preclinical research

## Abstract

Skin conductance monitoring is one of the promising methods for objectively evaluating pain. However, skin conductance might possibly increase in response to sympathetic stimulation other than pain. In this study, we aimed to test whether skin conductance monitoring can distinguish physical pain stimulation (heat, mechanical and cold stimulation) from other sympathetic stimuli (stimulation by noise and painful images). Twenty-three healthy volunteers participated in this prospective observational study. The number of fluctuations in skin conductance (NFSC) and normalized skin conductance level (nSCL) were measured and compared with pain scores on a self-reported pain scale (numerical pain scale [NPS]). Both NFSC and nSCL increased during mechanical stimulation. Further, nSCL, but not NFSC, well reflected heat stimulus intensity, suggesting its ability to quantitatively evaluate pain. nSCLs during physical pain stimulation were greater than those during other sympathetic stimulations. However, NFSC was not able to completely distinguish between the stimuli. These results suggest that nSCL could better differentiate physical pain stimuli from other sympathetic stimuli than NFSC. In comparisons between subjective and objective pain assessment in the same individual, nSCL correlated better with NPS score, indicating the possibility of being able to monitor the transition of pain. Monitoring changes in skin conductance using nSCL might be useful for objectively detecting physical pain.

## Introduction

Psychometric response scales, such as the visual analogue scale, have been widely used for assessment of pain^1^. This seems to be reasonable because the sensation of pain is usually affected by psychogenic factors, including mood, attention, anxiety, expectation, hypnosis and empathy^2^. This fact is reflected in the following definition of pain by the International Association for the Study of Pain: An unpleasant sensory and emotional experience with actual or potential tissue damage, or described in terms of such damage^3^. However, an objective pain assessment tool is still required, because verbal expression of pain is difficult for certain individuals, such as children, mentally-handicapped persons and intubated and sedated patients.

Numerous studies regarding objective pain assessment have been conducted, and several physiological markers of pain, such as heart rate variability and pupillary reflexes, were proposed. However, none of them has been established as a validated marker for pain assessment so far^4,5^. Among the different markers proposed, changes in skin conductance might be promising. Activation of sympathetic nerves in the skin following the experience of pain sensation and/or certain emotions, including fear and excitement, results in sweating in the palmar and plantar areas, which in turn increases skin conductance in these areas. Hence, the rationale for development of the skin conductance monitor was to detect changes in skin conductance over time^6^.

The principle of the skin conductance monitor raised the question regarding whether it could be used as a pain monitor. In other words, whether the skin conductance monitor can distinguish a stimulus that stimulates a sympathetic nerve but does not cause pain from a stimulus that causes pain. Few studies have, however, aimed to clarify this issue.

In most studies using the skin conductance monitor, the number of fluctuations in skin conductance (NFSC) was evaluated. Indeed, NFSC reportedly increased in response to painful stimuli^7,8^ and well reflected pain intensity^9–14^, but not in other studies^15–18^. These results suggested that NFSC is not necessarily useful for the assessment of pain. On the other hand, the normalized skin conductance level (nSCL) has been used to discriminate pain intensity in several studies. In one study, nSCL was reportedly even better than NFSC^19^.

Based on this evidence, we hypothesized that it might be possible for the skin conductance monitor to discriminate between physical pain stimuli and other sympathetic stimuli by using nSCL rather than NFSC as the assessment parameter. To investigate this hypothesis, we conducted the present study on healthy volunteers who were exposed to various experimental stimuli. Further, as already shown by previous studies^19,20^, we examined whether the skin conductance monitor can differentiate various thermal pain intensities.

## Material and methods

### Participants

This study was approved by the institutional review board of Gunma University Graduate School of Medicine (Approval No. 1031) and was registered with the University Hospital Medical Information Network Clinical Trials Registry (UMIN-CTR ID: 000,014,614). The study was conducted between February 2014 and October 2015 in accordance with institutional ethics provisions and the Declaration of Helsinki. The participants in the present study were 23 healthy volunteers, including 14 women and 9 men, ranging in age from 20 to 48 years old. Written informed consent was obtained from each participant. Inclusion criteria were: (1) age between 20 and 60 years, and (2) ability to understand the purpose and instructions of the study. Exclusion criteria were: (1) suffering from any type of chronic pain; (2) inability to tolerate a heat stimulus; (3) use of medication(s) that can potentially affect the activity of the autonomic nervous system; (4) not suitable for receiving a physical pain stimulus because of severe pre-existing disease; and (5) pregnancy.

The sample size of this study was determined by the results of our pilot study, in which a total of six subjects participated. In the pilot study, we applied stimuli at 32 °C and 47 °C in random order. As a result, the mean difference in nSCL values and averaged SD values of nSCL after application of the 32 °C and 47 °C stimuli was determined to be 351 and 460, respectively. The standardized mean difference was 0.75. From this value, a significance level of 0.05 and a power of 80%, the required sample size was calculated as 16 subjects. Anticipating a drop-out rate of up to 20% because the intervention in this study might have been painful, 23 individuals were recruited to the study.

### State-Trait Anxiety Assessment

We measured the degree of anxiety in participants before the training session by using the State-Trait Anxiety Inventory-Form JYZ (STAI-JYZ) questionnaire, because we considered the possibility that anxiety might affect the test results^9,21^. The questionnaire we used was the Japanese version of STAI (Form Y) for measurement of emotional states (STAI Y-1) and personality traits (STAI Y-2). The original version of the questionnaire was developed by Gaudry et al.^22^, following which the Japanese version was published^23^. Since each questionnaire consists of 20 questions and each question is allotted 1–4 points, the total scores of each questionnaire range from 20 to 80 points. Although assessment of the degree of anxiety in participants during/after painful events is suggested to be important ^9^, such assessments were not performed in this study.

### Study procedure

All tests were performed by a single tester in the same room of Gunma University Hospital. We monitored the temperature of the room used for the experiments and confirmed that it was tightly controlled (25.3 ± 1.1 °C).

### Training session

All the participants were trained to evaluate pain intensity before the main tests. The participants were asked to assess pain on a numerical pain scale (NPS: from 0 = no pain to 100 = the worst pain imaginable), which they expressed verbally at the end of each stimulus period. In the training session, a thermode of 30 × 30 mm (Pathway Model ATS, Medoc Ltd., Ramat Yishai, Israel) was attached to the volar forearm on the dominant hand side, and heat stimuli were applied through the thermode. The thermode temperature was gradually raised from 32 °C (8 °C/s) to reach the target temperature (45, 46, or 47 °C) within 2 s, and was maintained at this temperature for 7 s. Each target temperature was chosen once in random order. This training session was repeated three times at 1 min intervals.

### Main tests

**Figure 1:**
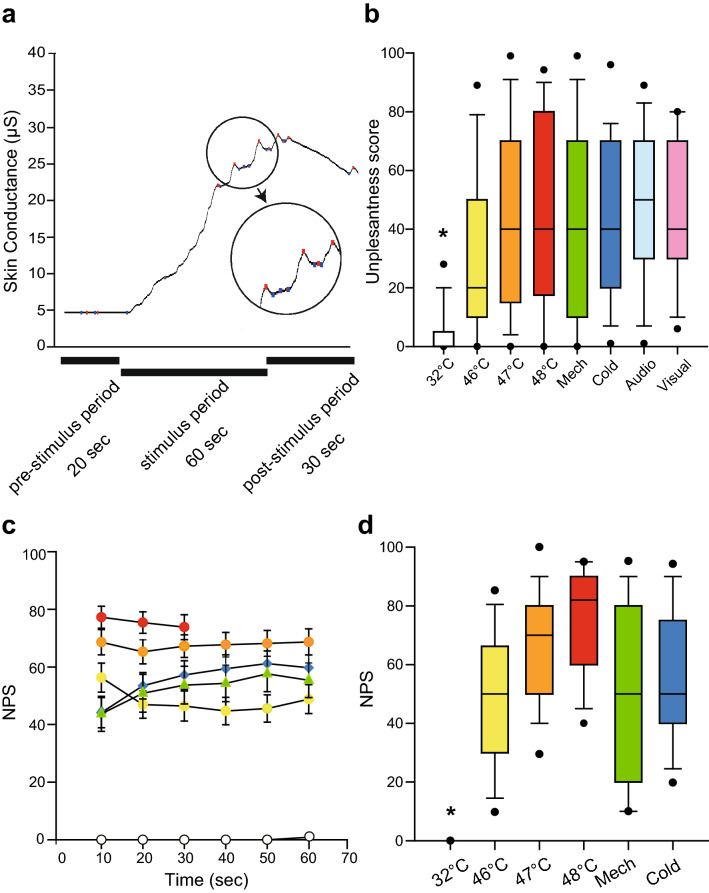
Examples of changes in skin conductance and the subjective evaluation of various stimuli. (**a**) Sample changes in skin conductance. Skin conductance was stable in the pre-stimulus period, but significantly increased in the stimulus period, with several peaks as shown in the circle. Each peak consisted of minimum and maximum values, marked with blue and red dots, respectively. (**b**) Comparison of unpleasantness scores between control stimulation and all other types of stimuli (i.e. heat, mechanical, cold, auditory and visual stimuli). The box shows the 25th percentile, median and 75th percentile. Error bars above and below the box indicate the 90th and 10th percentiles, respectively, and the black dots above and below the error bars are the 95th and 5th percentiles, respectively. Unpleasantness scores during control stimulation (32 °C) were lower than those during all other stimulations. One-way ANOVA post hoc Student–Newman–Keuls test, * *P* < 0.05. (**c**) Time course of numerical pain scale (NPS) scores during the stimulation period. (**d**) Comparison of NPS scores during the stimulation period. NPS scores during control (32 °C) stimulation were significantly different from NPS scores during all other stimulations. One-way ANOVA post hoc Student–Newman–Keuls test, *P* < 0.05, * 32 °C vs. all other stimuli, Mech: mechanical, Audio: auditory.

All the participants were exposed to physical pain stimuli (heat, mechanical and cold) and other sympathetic stimuli (noisy auditory stimulus and visual stimulus that evoked the thought of pain). The participants drew lots to determine the order of presentation of these five categorized stimuli, so that the order of stimulation was random, and the participants were blinded to the order in which the stimuli would be presented. Each stimulus session involved waiting (5–10 min), pre-stimulus (20 s), stimulus (60 s) and post-stimulus periods (30 s) (Fig. 1a).

To obtain stabilized values of skin conductance before each stimulus, participants were asked to wait for 5–10 min while wearing noise-cancelling headphones (BOSE, QuietComfort 15) and closing their eyes (waiting period). Participants were asked to open their eyes 20 s before presentation of each stimulus (pre-stimulus period). All stimulations lasted for 60 s (stimulus period). After exposure to the stimulus, the participants were asked to remain still for 30 s (post-stimulus period). During the physical pain stimulus period (i.e., heat, mechanical and cold stimuli), participants were asked to verbally rate pain intensity using the NPS every 10 s. For all stimuli, participants were also asked to verbally rate unpleasantness on a numerical scale (from 0 = not unpleasant at all to 100 = extremely unpleasant) after the post-stimulus period, to evaluate the emotional impact of each stimulus^20^. Pain intensity and unpleasantness refer to the sensory-discriminate component and emotional-motivational component of pain, respectively.

### Stimuli

For heat stimulation, three temperatures were adopted: 46 °C, 47 °C and 48 °C. We adopted multiple stimuli of different temperatures because a secondary aim of this study was evaluation of the ability of the skin conductance monitor to assess quantitative pain. The same equipment was used as in the training session. Although the stimulus period was 60 s, as with the other stimuli, the stimulus period for 48 °C was shortened to 30 s to avoid burn injury, in accordance with the users’ manual. The order in which the target temperature was applied was determined by additional lottery draws by the participants. For convenience, the control stimulus of 32 °C was included in the heat category. Hence, the lottery was used to determine the order of stimulation by four targeted temperatures.

For mechanical stimulation, the tester applied pressure on the inter-digital web between the second and third fingers of the non-dominant hand, using an algometer to which a 1 cm^2^ rubber tip was attached (Somedic AB, Stockholm, Sweden)^24^. The pressure was increased from 0 to 250–300 kPa at a rate of 30 kPa/s.

For cold stimulation, we applied frozen ice packs (size, 12 × 6 cm) on the medial aspect of each participant’s non-dominant forearm^25–27^.

For auditory stimulation, the participants continued wearing headphones that were connected to an audiometer (AA-76, Rion Co., Ltd., Tokyo), and were forced to listen to masking sounds (band noise, 1,000 Hz, 85 dB) generated by the audiometer.

For visual stimulation, an image of an 18 G needle penetrating the forearm was shown on a display placed in front of the participant^28^. The participants were asked to imagine that the arm on the display was their arm.

The volume of noise and the picture used for visual stimulation were determined so that the unpleasantness score was comparable between stimuli. In our pilot study, we confirmed that the unpleasantness score of all types of stimuli was comparable.

### Measurement and processing of skin conductance

Skin conductance was recorded using the Pain Monitor (Med-Storm, Oslo, Norway) with three circular electrodes (1-cm diameter). A sample recording of skin conductance is shown in Fig. 1a. The sampling rate was 65 Hz. Measuring electrodes, a counter current electrode, and a reference voltage electrode were positioned on the hypothenar and thenar eminences and below the middle finger of the dominant hand, respectively^6,29^. To measure and analyze skin conductance values, we developed our own software program for converting the graphic data acquired by the Pain Monitor to numerical values. SCL and NFSC were calculated as previously reported^19^. SCL was defined as the average skin conductance value (μS) for 10 s. SCL was normalized by calculating the percentage change from the pre-stimulus average: normalized SCL (nSCL) = 100 × (SCL − average SCL in the pre-stimulus period)/average SCL in the pre-stimulus period ^19^. Average SCL in the pre-stimulus period was defined as the averaged SCL of the 60 s just before asking the participant to open their eyes for preparation of the stimulus period. Skin conductance fluctuations were defined as peaks with minimum amplitudes of 0.02 μS and a slope rate < 2 μS/s^29^. The NFSC value was defined as the number of skin conductance fluctuations during the 10 s period.

### Analysis

Analysis was performed using Sigmaplot version 12.5 (Systat Software Inc., CA, USA). One-way analysis of variance (ANOVA) with post-hoc Student–Newman–Keuls test was used for comparison of unpleasantness scores, NPS, nSCL and NFSC among all types of stimuli. Pearson’s correlation coefficients between total STAI score and unpleasantness score were calculated to investigate the relationship between the degree of anxiety and subjective evaluations. Similarly, the relationship between total STAI score and NPS was also examined.

Receiver operating characteristic (ROC) curve analysis was performed to investigate the cut off value, sensitivity and specificity of skin conductance variables in distinguishing each physical pain stimulus from other sympathetic stimuli. Moreover, the area under the ROC curve of nSCL and NFSC was compared. For the comparison between NPS and skin conductance values (i.e. nSCL and NFSC), non-parametric Spearman rank order correlation analysis was performed because some of these data were not normally distributed.

## Results

### Participants

Although one participant declined exposure to the 48 °C heat stimulus because of unendurable pain, the remaining 22 participants were able to complete the study. There were no severe complications, including heat injury with blisters, throughout the study in any of the participants. The participants’ characteristics are shown in Table 1.

**Table 1:** Characteristics of participants included in this study and results of the State-Trait Anxiety Inventory (STAI).

	Number	Age (year)	STAI score	
			State anxiety	Trait anxiety
Male	9	34.8 ± 10.3	29.2 ± 5.3	32 ± 4.8
Female	14	23.5 ± 4.3	31.3 ± 5.5	34.2 ± 5.5
Total	23	28.0 ± 9.3	30.5 ± 5.4	33.3 ± 5.3

All values are expressed as the mean ± SD.

### Subjective evaluation of the stimuli

Unpleasantness scores during each stimulus are shown in Fig. 1b. The only difference in unpleasantness scores was that between control (32 °C) stimulation and all other stimuli (*P* < 0.05, one-way ANOVA with post-hoc Student–Newman–Keuls test). These results suggest that the emotional impact of these stimuli, except for the control stimulus, was comparable.

Verbal responses (i.e. NPS scores) during physical pain stimulation are shown in Fig. 1c and d. Since stimulation at 48 °C was applied for only 30 s, it was excluded from statistical analysis. The only difference in NPS scores was that between control (32 °C) stimulation and all other stimuli (Fig. 1d, *P* < 0.05, one-way ANOVA with post-hoc Student–Newman–Keuls test). These results demonstrated that there was no difference in subjective evaluation of physical pain stimuli (heat, mechanical, and cold stimuli).

### Effect of anxiety on subjective evaluation

We next investigated the relationship between anxiety scores and subjective evaluation of stimuli. The degree of anxiety in all participants was assessed by the STAI before the experiment (Table 1). STAI scores (anxiety scores) did not correlate with unpleasantness scores (47 °C heat; r = 0.03, mechanical; r =  − 0.03, cold; r =  − 0.05, audio; r =  − 0.07, visual; r = 0.35). Moreover, STAI scores did not correlate with NPS scores (47 °C heat; r = 0.008, mechanical; r = 0.01, cold; r = 0.15). These results suggest that anxiety status did not appear to affect the subjective evaluation of unpleasantness or subjective pain evaluation.

### Objective evaluation of stimuli

**Figure 2:**
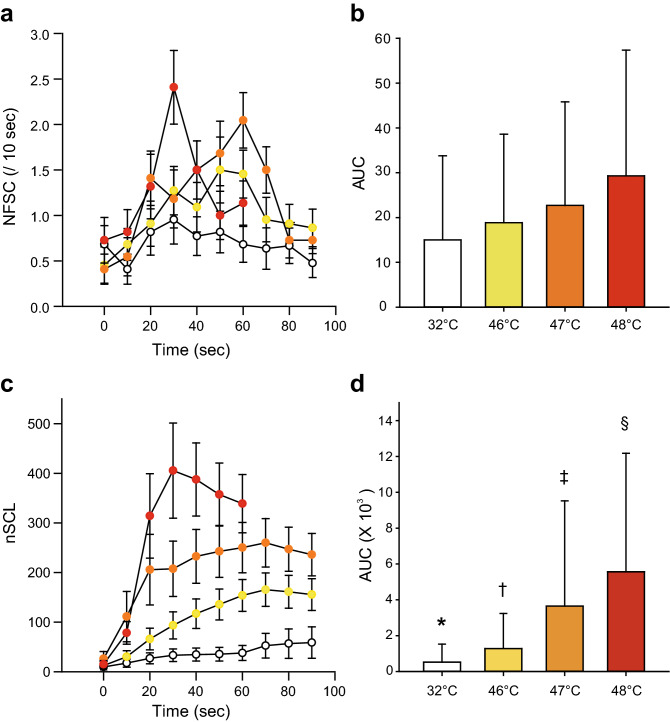
Changes in skin conductance during the heat stimulus period. (**a**) Time course of NFSC during heat stimulation. The white, yellow, orange and red circles indicate NFSC values during control (32 °C), 46 °C, 47 °C and 48 °C heat stimulation. (**b**) Comparison of the area under the curve (AUC) of NFSC during heat stimulation with various temperatures indicated no significant differences between them. (**c**) Time course of nSCL during heat stimulation. nSCL reflected heat intensity well. (**d**) AUC of nSCL between the different temperatures used for heat stimulation. There were significant differences among all pairs of heat stimuli, including control (32 °C) stimulation. One-way ANOVA post hoc Student–Newman–Keuls test, *P* < 0.05, * 32 °C vs. all other stimuli, † 46 °C vs. all other stimuli, ‡ 47 °C vs. all other stimuli, § 48 °C vs. all other stimuli.

Several past studies suggested the superiority of nSCL to NFSC for differentiating heat intensities^19,20^. In order to validate the results of these past studies, heat stimuli at various temperatures (32, 45, 46 and 47 °C, 48 °C) were applied in this study, and NFSC and nSCL were compared. NFSC did not reflect the temperature of heat stimuli (Fig. 2a). There were no significant differences in the area under the curve between the different heat stimulus temperatures (Fig. 2b, *P* > 0.05, one-way ANOVA post hoc Student–Newman–Keuls test). nSCL on the hand well reflected heat intensity (Fig. 2c), with significant differences in nSCL between control stimulation and each heat stimulus temperature (*P* < 0.05, one-way ANOVA post hoc Student–Newman–Keuls test, Fig. 2d).

**Figure 3:**
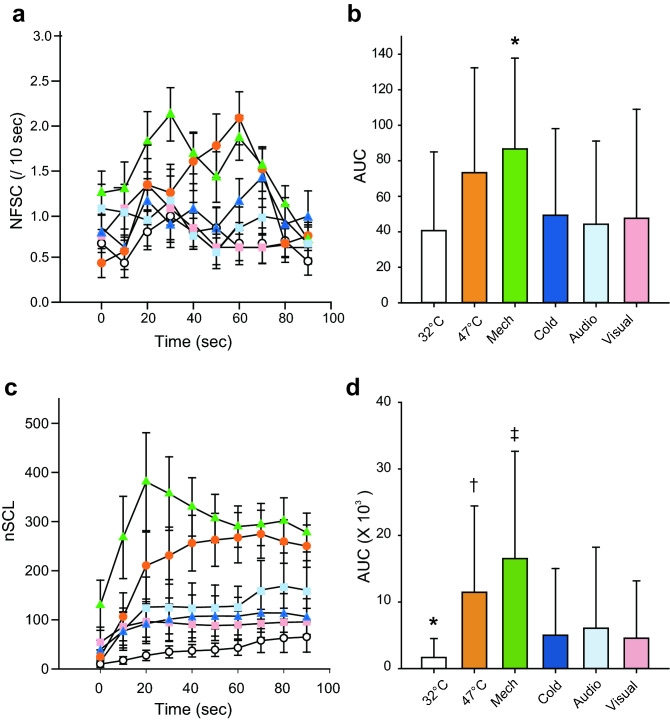
Changes in skin conductance during physical pain stimulation and other sympathetic stimulation. (**a**) Time course of NFSC during all types of stimulation. The white and orange circles indicate NFSC values during control (32 °C) and 47 °C heat stimulation. The green and blue triangles indicate NFSC values during mechanical and cold stimulation, and light blue and pink squares indicate NFSC values during auditory and visual stimulation. (**b**) Area under the curve (AUC) of NFSC during all types of stimulation. (*P* < 0.05,). Although AUC with mechanical stimulation was significantly different compared to all other stimuli, there was no significant difference between other pairs of stimuli. One-way ANOVA post hoc Student–Newman–Keuls test, * *P* < 0.05. (**c**) Time course of nSCL during all types of stimulation. (**d**) AUC of nSCL during all types of stimulation. There were significant differences in AUC of nSCL between the common physical pain stimuli (i.e. heat and mechanical stimuli) and other sympathetic stimuli (i.e. auditory and visual stimuli). One-way ANOVA post hoc Student–Newman–Keuls test, *P* < 0.05, † 47 °C vs. cold, auditory, and visual stimuli, ‡ mechanical vs. cold, auditory, and visual stimuli. AUC of nSCL during control (32 °C) stimulation was significantly smaller than that during other physical pain stimuli. One-way ANOVA post hoc Student–Newman–Keuls test, * *P* < 0.05. Mech: mechanical, Audio: auditory.

Next, we examined whether skin monitoring can distinguish the physical pain stimulus from other sympathetic stimuli. Changes in NFSC during all types of stimulation are shown in Fig. 3a. NFSC was not able to distinguish the physical pain stimulus (i.e. 47 °C heat, mechanical and cold stimuli) from other sympathetic stimuli (i.e. noise and visual stimuli), as shown in Fig. 3a. The only exception was NFSC after mechanical stimulation. NFSC during mechanical stimulation was greater than that during other stimuli (Fig. 3b, *P* < 0.05, one-way ANOVA post hoc Student–Newman–Keuls test). Changes in nSCL during all types of stimulation are shown in Fig. 3c. nSCL during control (32 °C) stimulation was smaller than that during application of any other stimuli (Fig. 3c, d, *P* < 0.05, one-way ANOVA post hoc Student–Newman–Keuls test). Moreover, nSCLs during physical pain stimulation (heat and mechanical stimuli) were greater than those during other sympathetic stimulations (Fig. 3c, d, *P* < 0.05, one-way ANOVA post hoc Student–Newman–Keuls test). These results suggest that nSCL could better differentiate the common physical pain stimuli (mechanical and heat stimuli) from other sympathetic stimuli than NFSC.

### Receiver operating curve analysis

**Figure 4:**
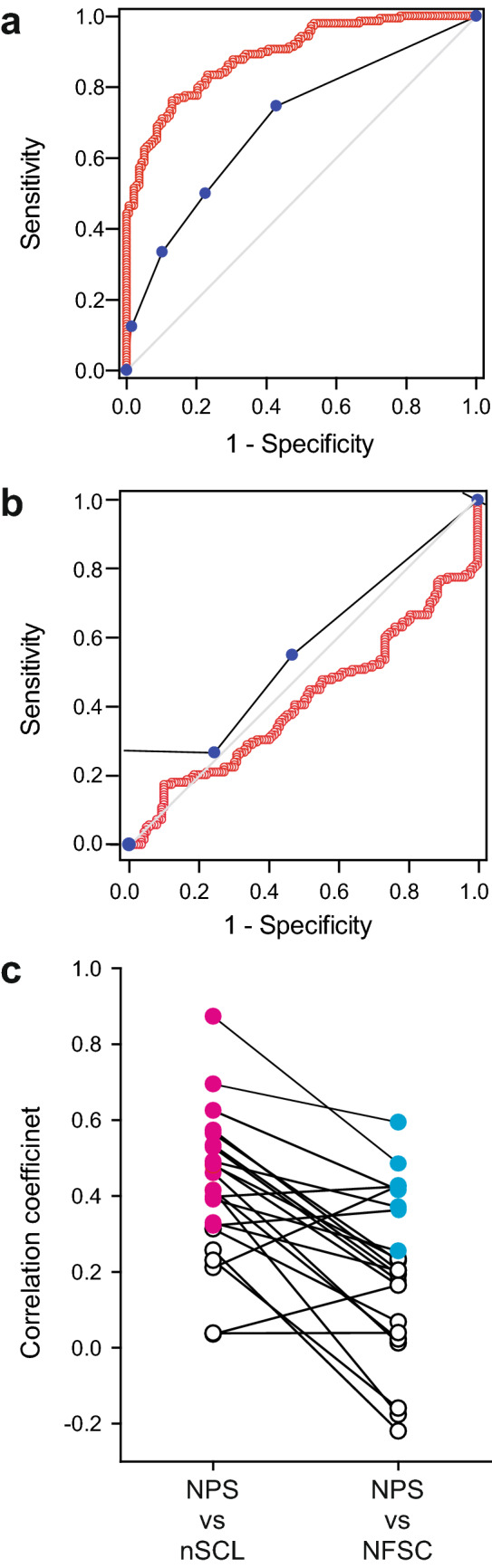
Sample receiver operating characteristic (ROC) curves for discrimination between physical pain stimuli and other sympathetic stimuli in terms of the number of fluctuations in skin conductance (NFSC) and normalized skin conductance level (nSCL) (**a**, **b**). The red open circles and blue filled circles indicate nSCL and NFSC, respectively. The gray diagonal line represents the results of random guessing. (**a**) ROC curves for mechanical stimulation as the physical pain stimulus and visual stimulation as representative of the other sympathetic stimuli. Although both nSCL and NFSC could differentiate the physical pain stimulus from other sympathetic stimuli, nSCL showed better discrimination ability than NFSC (*P* < 0.001). (**b**) ROC curves for the cold stimulus as the physical pain stimulus and auditory stimulus as representative of the other sympathetic stimuli. Both nSCL and NFSC could not differentiate physical pain stimulation from other sympathetic stimuli. (**c**) Correlation coefficients between numerical pain scale (NPS) scores and skin conductance for each individual. Each dot indicates the correlation coefficient for each participant. Spearman’s rank correlation analysis indicated that nSCL correlated with NPS better than did NFSC (*P* < 0.001). The magenta filled circles indicate a significant correlation between intra-individual nSCL and NPS, whereas the cyan filled circles indicate a significant correlation between intra-individual NFSC and NPS. White open circles indicate non-significant correlations.

We performed ROC curve analysis to test whether NFSC and nSCL could discriminate between physical pain stimuli and other sympathetic stimuli. Sample ROC curves are shown in Fig. 4. Figure 4a shows ROC curves of NFSC and nSCL when mechanical and visual stimuli were compared, and Fig. 4b shows ROC curves of NFSC and nSCL when cold and auditory stimuli were compared. Table 2 presents a summary of ROC curve analysis. The accuracy for differentiating physical pain stimuli from other sympathetic stimuli was evaluated using area under the ROC curve analysis. The area under the ROC curve of NFSC was 0.6 or more for both heat (47 °C) and mechanical stimuli as compared to control (32 °C), auditory and visual stimuli. This was also true for nSCL. However, the area under the ROC curve of NFSC was smaller than 0.6 with cold stimuli as compared to control (32 °C), auditory and visual stimuli. Again, this was also true for nSCL. These results indicate that both NFSC and nSCL could discriminate common physical pain (heat and mechanical stimulation) from other stimuli (including control). However, neither NFSC nor nSCL could distinguish cold stimulation from other stimuli. Next, we compared NFSC and nSCL to determine their discriminatory ability. The gray cells in Table 2 show that the area under the ROC curve of nSCL was greater than that of NFSC (*P* < 0.05). This suggests that nSCL can distinguish between mechanical stimulation and stimulation without a painful sensation (control, auditory and visual stimuli) with high accuracy compared to NFSC. For heat stimulation (47 °C), the discriminatory superiority of nSCL was shown only in comparison with the control stimulus (Table 2).

**Table 2:** Results of receiver operating characteristic (ROC) curve analysis for discrimination between physical pain stimuli and other sympathetic stimuli by NFSC and nSCL.

		Control (32 °C)			Auditory			Visual		
		Cut off	Sensitivity Specificity	Area	Cut off	Sensitivity Specificity	Area	Cut off	Sensitivity Specificity	Area
47 °C	nSCL	21.84	0.70 0.70	**0.76**	68.97	0.56 0.58	0.60	39.69	0.64 0.63	0.66
	NFSC	0.50	0.59 0.57	**0.62**	0.50	0.59 0.54	0.60	1.5	0.42 0.78	0.60
Mech	nSCL	93.14	0.76 0.87	**0.89**	152.00	0.64 0.78	**0.77**	102.80	0.75 0.75	**0.80**
	NFSC	0.50	0.75 0.57	**0.70**	0.50	0.75 0.54	**0.68**	0.50	0.75 0.54	**0.68**
Cold	nSCL	22.21	0.48 0.70	0.59	–	–	0.41	–	–	0.48
	NFSC	0.5	0.55 0.57	0.56	0.50	0.55 0.54	0.54	0.5	0.55 0.54	0.54

nSCL could discriminate between common physical pain (heat and mechanical stimulation) and other types of stimuli (including control) significantly better than NFSC. In the stimulus combination where the values of the area under the curve are shown in bold, the area under the curve of nSCL is greater than that of NFSC (*P* < 0.05). Since the area under the curve of nSCL during cold stimulation was less than 0.5 when it was compared to that during both auditory and visual stimulation, we did not calculate cut off values, sensitivity and specificity for these comparisons. *Mech* mechanical.

### Correlation between subjective and objective evaluations

Correlation coefficients between subjective evaluations (unpleasantness score or NPS score) and changes in skin conductance values (NFSC or nSCL) were calculated to compare subjective and objective evaluations of pain. We found no significant correlation between unpleasantness score and skin conductance value (data not shown). There was a weak correlation (r = 0.28) between NPS and nSCL scores only for the mechanical stimulus (Table S1).

In addition to the analyses using all population data, correlation coefficients between NPS and skin conductance (NFSC or nSCL) for each individual were calculated. This was done because although the correlation between NPS and skin conductance (NFSC or nSCL) was poor in the analysis using the entire population data (Table S1), we speculated that this poor correlation might be due to a large inter-participant variability in skin conductance responses.

Intra-individual analysis showed that nSCL correlated with NPS in 16 out of 23 participants (70%), whereas NFSC correlated with NPS in only eight out of 23 participants (35%), as shown in Fig. 4C. The correlation coefficients between NPS and nSCL were greater than those between NPS and NFSC (Fig. 4c, *P* < 0.001, Spearman rank order correlation analysis).

## Discussion

We showed here that both the skin conductance parameters, NFSC and SCL, can differentiate between physical pain stimuli (except the cold stimulus) and other sympathetic stimuli, although the accuracy was higher with nSCL than NFSC. Moreover, nSCL showed particularly good agreement with subjective pain assessment in individual analysis.

The purpose of this study was to test whether skin conductance monitoring can distinguish between stimuli that stimulate the sympathetic nerves but do not cause pain and those that cause pain as well as stimulate these nerves. We measured unpleasantness scores and confirmed that their values during pain stimulation were comparable to those with other sympathetic stimulation (Fig. 1b), suggesting similar emotional impacts of each stimulus.

Skin conductance, especially nSCL, was found to be able to distinguish between physical pain stimuli and other sympathetic stimuli. Painful stimuli seem to naturally accompany various other stimuli, including tactile and mental stimuli. Although the skin conductance monitor indicated a response even to stimuli that did not cause pain, the degree of the reaction was weaker than the response to painful stimuli. We believe that the skin conductance monitor can be used as a pain-specific monitor. The mild increase in nSCL after stimulation with auditory and painful visual images support this idea (Fig. 3).

Elevation of nSCL during cold stimulation was smaller than that with other physical pain stimuli (Fig. 3d), although subjective evaluation showed that pain due to cold stimulation was equivalent to that of other pain stimuli (i.e., heat and mechanical). Although there was no difference in subjective pain assessment when comparing heat and cold stimulation, muscle sympathetic nerve activity was higher during heat stimulation as compared to cold stimulation in a previous study^30^. Although this result is consistent with our research results, it is necessary to investigate methods of objective pain assessment during cold stimulation. From another point of view, the area in contact with the skin when the ice pack for cold stimulation was pressed against the skin was greater than that for heat and mechanical stimulation. These differences in the size of the area over which the stimulus was applied might have affected changes in skin conductance. In addition, since there is no definite view on the transmission pathway of skin sensations that eventually stimulate the sympathetic nerves and produce the resultant biological responses, including sweating, further research on this is necessary.

Most research on the evaluation of pain using skin conductance monitors first categorized the degree of pain and then determined the threshold of NFSC in each category^10,15,16,19^. However, since we observed changes in skin conductance with various experimental stimuli, we determined thresholds according to the type of stimulation, and not the degree of pain. In order to directly compare our results with those of previous studies that first categorized the degree of pain, we examined the degree of pain experienced by the participants with each stimulus. In particular, we referred to the paper by Treister et al. for comparison^19^. They adjusted the stimulation temperature for each individual so that NPS score would fall into three categories (“low pain”: ~ 30, “medium pain”: ~ 60 and “high pain”: ~ 90). As a result, “medium pain”, with an NPS score of 53.5 ± 18.9, was generated by a heat stimulus of 47.43 ± 0.63 °C, and “high pain”, with an NPS score of 70.2 ± 18.9, was generated by heat stimulation at a temperature of 48.34 ± 0.33°C^19^. In the present study, pain with NPS scores of 48.13 ± 24.18 and 67.62 ± 21.04 were generated by heat stimulation at temperatures of 46 °C and 47 °C, respectively. These results suggest that heat stimulation at 47 °C in this study is considered to have caused pain corresponding to “high pain”. It is unknown why the participants of this study expressed NPS scores equivalent to “high pain” at lower temperatures (i.e. 47 °C) than in the previous study. Treister et al. reported that both NFSC and SCL could distinguish between “high pain” and “no pain” to the same extent (*P* < 0.001), but SCL rather than NFSC could more accurately distinguish pain intensity on the whole^19^. These results are consistent with our results. However, since they did not evaluate pain intensity by determining the pain threshold, we further compared our results with other studies that analyzed the responses in a similar manner as our method. Since our participants did not include children, our results were compared to those in adults. In a previous study on postoperative pain, when no pain to mild pain (score 0–3) and moderate to severe pain (score 4–10) were distinguished by setting the threshold of NFSC to 0.1, the sensitivity and specificity was reportedly 88.5% and 67.7%, respectively^10^. However, in the case of awake patients as compared to immediate post-anesthesia patients, other stresses might be included in skin responses judged to be less severe than moderate pain (score < 4–5)^31^. In the present study, absence of sedation in the participants might have affected the assessment of pain using NFSC.

Use of NFSC assessment is advantageous over nSCL evaluation, since devices for evaluating pain using NFSC are commercially available. Further, many studies have shown that NFSC assessment can be used to evaluate pain in patients of all ages and in various clinical settings, including postoperative and intensive care units^9–11,15–18,32,33^. In this study, however, nSCL had a better correlation with subjective pain assessment compared to NFSC within the same individual (Fig. 4c), indicating that nSCL might be useful for monitoring the transition of pain in each individual. This is consistent with the results of a previous study ^16^, in which the authors argued that nSCL did not significantly correlate with each participant’s pain rating at the group level despite the strong within-participant correlations. Although this is not a new way of standardization^19,20^, obtaining a “stimulation mean” and “prestimulation mean” for calculating changes in skin conductance might help to minimize inter-individual differences. Further investigations to test whether nSCL can evaluate pain in clinical practice will be needed in the future, because studies using nSCL, including the present study, targeted experimental pain^19^.

Self-reported pain is influenced by several factors, including previous pain experiences, gender, cultural background, sociodemographic factors, anxiety and depression^12^. For example, preoperative anxiety has been reported to affect postoperative pain^21^. Further, in a study on experimental pain, the anxiety level reportedly correlated with the numerical rating scale of pain^9^. In this study, however, anxiety status did not correlate with subjective pain evaluation. This discrepancy might have resulted from the fact that only a few of the participants in our study were anxious. Alternatively, the difference in the evaluation method of anxiety level might have influenced the results. For example, anxiety status in our participants was measured only at the beginning of the experiment, although it could have changed during the course of the study. Lack of examination of anxiety status when the stimuli were applied is a limitation of this study.

Since skin temperature reportedly correlates with the skin conductance value^33^, lack of skin temperature monitoring might be another limitation of this study. However, since we expect that changes in the participants’ skin temperatures during the experiments were not significant, we believe that the influence of skin temperature on the skin conductance value in this study was minimal.

Several researchers have shown the efficacy of objective pain evaluation using multiple physiological parameters, including “nociception level index”^34^. Indeed, devices using four sensors, namely a photoplethysmograph, galvanic skin response, temperature and accelerometer, are already commercially available for use in a clinical setting^5,34–36^. The results of the present study suggest that nSCL should also be considered as one of the many parameters that can be used in such monitors. In future, since skin conductance monitoring is minimally invasive, it might become a common tool for monitoring pain in the clinical setting.

## Conclusion

Although both NFSC and nSCL increase during mechanical stimulation, only nSCL increases during heat stimulation. This study showed that nSCL is more useful for the detection of physical pain than NFSC. Skin conductance monitors appear to be useful for detecting physical pain in the clinical setting.

## Supplementary information

Supplementary file1 (PDF 131 kb)

## Data Availability

The datasets of the current study are available from the corresponding author on reasonable request.
