# Supplementary material for: Normalized skin conductance level could differentiate physical pain stimuli from other sympathetic stimuli
Source: Sci Rep. 2020 Jul 2;10:10950. doi: 10.1038/s41598-020-67936-0 (PMC7331661; doi:10.1038/s41598-020-67936-0)
Supplement: Supplementary file 1 — Supplementary file1 (PDF 131 kb) [file 41598_2020_67936_MOESM1_ESM.pdf]

Supplementary information

Normalized skin conductance level could differentiate physical pain stimuli from other sympathetic stimuli

Satomi Sugimine[1], Shigeru Saito[1,2], and Tomonori Takazawa[1]

[1] Intensive Care Unit, Gunma University Hospital, 3-39-15 Showa-machi, Maebashi, Gunma 371-8511, Japan

[2] Department of Anesthesiology, Gunma University Graduate School of Medicine, 3-39-22 Showa-machi, Maebashi, Gunma 371-8511, Japan

Corresponding author

Tomonori Takazawa, MD, PhD

Mailing address: 3-39-15, Showa-machi, Maebashi, Gunma 371-8511, Japan

Tel: +81-27-220-8698; Fax: +81-27-220-8692

E-mail: takazawt@gunma-u.ac.jp

Table S1. Correlation coefficients between numerical pain scale (NPS) scores and skin conductance values

		NPS					
		Control (32°C)	46°C	47°C	48°C	Mech	Cold
nSCL	Control (32°C)	r = 0.003<br>p = 0.97					
	46°C		r = -0.030<br>p = 0.73				
	47°C			r = 0.14<br>p = 0.11			
	48°C				r = 0.076<br>p = 0.54		
	Mech					r = 0.28<br>p < 0.001	
	Cold						r = -0.05<br>p = 0.58
NFSC	Control (32°C)	r = -0.071<br>p = 0.41					
	46°C		r = -0.029<br>p = 0.74				
	47°C			r = 0.025<br>p = 0.77			
	48°C				r = -0.13<br>p = 0.29		
	Mech					r = -0.066<br>p = 0.44	
	Cold						r = -0.051<br>p = 0.55

Although there was a weak correlation between NPS and normalized skin conductance level (nSCL) for the mechanical stimulus, there was no correlation for the other stimuli. Mech: mechanical.
